# Green Synthesis, Characterization, and Antiparasitic Effects of Gold Nanoparticles against *Echinococcus granulosus* Protoscoleces

**DOI:** 10.3390/tropicalmed8060313

**Published:** 2023-06-09

**Authors:** Yosra Raziani, Pegah Shakib, Marzieh Rashidipour, Koroush Cheraghipour, Javad Ghasemian Yadegari, Hossein Mahmoudvand

**Affiliations:** 1 Nursing Department, Al-Mustaqbal University College, Hillah 51001, Babylon, Iraq; 2 Razi Herbal Medicines Research Center, Lorestan University of Medical Sciences, Khorramabad 6718773654, Iran; 3 Food and Drug Deputy, Lorestan University of Medical Sciences, Khorramabad 6718773654, Iran; 4 Molecular and Cellular Laboratory, School of Allied Medicine, Lorestan University of Medical Sciences, Khorramabad 6718773654, Iran; 5 Department of Medical Parasitology and Mycology, Lorestan University of Medical Sciences, Khorramabad 6718773654, Iran

**Keywords:** gold, nanomedicine, hydatidosis, echinococcosis, apoptosis

## Abstract

Echinococcosis, or hydatidosis, is one of the most important zoonotic diseases, which is initiated by the larval stage in the clasts of *Echinococcus granulosus*. For the treatment of hydatidosis, surgery is still the preferred method and the first line of treatment for symptomatic patients. Unfortunately, most of the scolicidal agents that are injected inside cysts during hydatid cyst surgery have side effects, including leaking out of the cyst and adverse effects on the living tissue of the host, such as necrosis of liver cells, which limits their use. This work was carried out to study the lethal effect of green synthesized gold nanoparticles (Au-NCs) against hydatid cyst protoscoleces. Au-NCs were green synthesized using the *Saturja khuzestanica* extract. Au-NCs were characterized by UV-visible absorbance assay, electron microscopy analysis, X-ray diffraction (XRD), and Fourier transform infrared (FTIR) spectroscopy. Scolicidal properties of Au-NCs (1–5 mg/mL) were studied against protoscoleces for 10–60 min. The effect of Au-NCs on the expression level of the caspase-3 gene as well as the ultrastructural examination was studied by real-time PCR and scanning electron microscopy (SEM). The cytotoxicity of Au-NCs on hepatocellular carcinoma (HepG_2_) and normal embryonic kidney (HEK293) cell lines was also studied by the cell viability assay. The obtained Au-NCs are cubes and have an average size of 20–30 nm. The highest scolicidal efficacy was observed at 5 mg/mL with 100% mortality after 20 min of treatment for hydatid cyst protoscoleces. In ex vivo, Au-NCs required more incubation time, indicating more protoscolicidal effects. Au-NCs markedly upregulated the gene level of caspase-3 in protoscoleces; whereas they changed the ultra-structure of protoscoleces by weakening and disintegrating the cell wall, wrinkles, and protrusions due to the formation of blebs. We showed the effective in vitro and ex vivo scolicidal effects of Au-NCs against hydatid cyst protoscoleces by provoking the apoptosis process of caspase-3 activation and changing the ultrastructure of protoscoleces with no significant cytotoxicity against human normal cells. However, additional studies should be conducted to determine the possible harmful side effects and accurate efficacy.

## 1. Introduction

In the science of nanotechnology, the production of different nanomaterials is applied in various fields such as medicine (drug targeting, imaging, and biosensors), food sciences, and environmental sciences [1]. Nanoparticles are compounds whose particle size reaches a maximum of one hundred nanometers, which is one of their unique characteristics. These particles have a great diversity in size and morphology, and as the particle size decreases, their surface-to-volume ratio increases [2]. This feature makes their special surface to carry out catalytic reactions and effects increase biologically among nanoparticles, and gold nanoparticles (Au-NCs) due to having low toxicity, high stability, and catalytic activity display various biological capabilities in medicine such as antibacterial, antiviral, antifungal, and antimalarial properties [2]. In the past, various synthetic approaches have been used to produce nanoparticles [3]. The production of nanoparticles using conventional approaches has low and difficult performance, requires a lot of temperature, pressure, and time, and is expensive; therefore, nowadays biological methods are used to produce nanoparticles [4]. Biosynthesis, as an environmentally friendly and cost-effective approach that uses bacteria, fungi, and plant extracts, has the potential to produce and synthesize nanoparticles [5]. The use of herb extracts to produce nanoparticles, which is also called “green synthesis”, makes the synthesis process easier and its progress faster [6]. The use of plant extracts in the synthesis of metal nanoparticles, especially gold, is very promising, and so far, different plants such as cinnamon, licorice, black tea, and eucalyptus have been used to synthesize gold nanoparticles [7].

Echinococcosis, or hydatidosis, is one of the most important zoonotic diseases, which is initiated by the larval stage in the clasts of *Echinococcus granulosus* [8]. The disease is endemic in sheep and cattle-raising regions worldwide. However, its prevalence is very high in the Mediterranean region, such as in Iran [8]. Excretion of eggs from adult worms causes soil and water pollution, and domestic animals such as sheep, goats, cows, and camels (intermediate hosts) eat eggs while grazing. Humans accidentally eat food or water contaminated with eggs, or they are infected with the intermediate stage of the parasite through direct contact with infected dogs. The swallowed parasite penetrates through the intestine and is transferred through the blood system to vital organs, e.g., the liver and lungs [8]. In areas with high prevalence, children are often affected, but liver cysts need several years to grow so large that they can be detected or have symptoms [8]. There is a possibility that all organs can be affected by this disease, but the most common site of infection is the liver (60 to 70%). Less than 2% of the reported cases are cysts in the brain, and other organs such as the spleen, muscle, and multi-organ disease of the liver and lung, or liver and spleen-lung and brain, have also been reported. Rarely, hydatid cysts have been reported in the heart, thyroid, breast, kidney, and soft tissue of the neck and vertebrae [9,10,11]. For the treatment of hydatidosis, surgery is still the preferred method and the first line of treatment in symptomatic patients [12]. Surgery is usually completed with anti-parasitic drugs, and in non-surgical cases, drug therapy is the only method of choice [13]. One of the measures to make cyst surgery safe is the use of a suitable scolicidal injection of hypertonic nitrate salt, formalin, hypertonic saline, silver nitrate, and cetrimide into the cyst [14]. Unfortunately, most of these scolicidal substances that are injected inside cysts have side effects, including leaking out of the cyst and adverse effects on the living tissue of the host, such as necrosis of liver cells, which limits their use [15]. Even though some investigations have been conducted on the protoscolicidal and antihydatid cyst effects of various nanoparticles, such as silver nanoparticles, at concentrations varying between 1 and 4000 µg/mL, their outcomes have been different and sometimes opposing, which may affect the effectiveness of the nanoparticles due to the synthesis approach of the nanoparticles, the efficacy measurement method, and the method of application [16]. *Saturja khuzestanica* belongs to the Laminaceae family and is an endemic herb that is broadly grown in various parts of the world [17]. The herb showed various pharmacological and therapeutic properties in modern and traditional medicine, e.g., antinociceptive, anti-inflammatory, antioxidant, anticancer, anti-bacterial, antiparasitic, antiviral, and antifungal properties [17]. Therefore, this work was carried out for the first time to study the lethal effect of gold nanoparticles synthesized with an aqueous extract of *S. khuzestanica* against hydatid cyst protoscoleces.

## 2. Materials and Methods

### 2.1. Green Synthesis of Gold Nanoparticles (Au-NCs)

Aerial parts of *S. khuzestanica* were obtained from Kimia Daru Aflak Co. (Khorramabad, Iran). For extraction, water solvent was mixed with dried plant powder in a ratio of 1:10, and then the obtained combinations were placed on a shaker for 1 h (180 rpm). They were then exposed to an ultrasound bath (Wise Clean) for 40 min at 60 °C. The obtained extracts were concentrated using a vacuum rotary evaporator at 40 °C. The extract was kept in a dark container at refrigerator temperature until testing. To synthesize, the extract was mixed with tetrachloroauric acid (HAuCl4, Sigma-Aldrich, Taufkirchen, Germany), whereas the mixture color shifted from yellow to dark red [18,19].

### 2.2. Characterization of Green Synthesized Au-NCs

#### 2.2.1. UV-Visible Analysis

UV-vis spectroscopy at 350–680 nm wavelength was utilized to measure the progress of the Au-NCs synthesis reaction. For this purpose, the absorption spectrum of a sample after dilution with deionized water at a ratio of 1:10 was measured in the visible light range with a UV-vis spectrophotometer from LSI-Alpha.

#### 2.2.2. Scanning Electron Microscope Analysis

The morphology of green synthesized Au-NCs was evaluated using a scanning electron microscope (SEM) (Hitachi Model S-4160) with 15 kV, magnification of 10×, and resolution of 1 nm.

#### 2.2.3. Analysis of X-ray Diffraction (XRD)

XRD crystallography of green synthesized Au-NCs was performed in the scan range of a common angle (10 to 80 °C). XRD diffraction was determined by measuring the Ka-ray source of a copper lamp with a wavelength of X beams in λ = 1.52 A0 by an XRD device model 2000 APD (GNR, Explorer, Rome, Italy).

#### 2.2.4. Fourier Transform Infrared (FTIR) Spectroscopy 

To study the chemical functional groups involved in the formation and stabilization of nanoparticles, dried Au-NCs (25 mg) with potassium bromide (KBr, 250 mg) were placed in an FTIR sample container and analyzed by an FTIR instrument (Agilent Technologies, Cary 630, Waldbronn, Germany) in the range of 400–4000 cm^−1^.

### 2.3. Preparing the Protoscoleces 

Liver hydatid cysts (>90% viability) were acquired from infected sheep provided in the Khorramabad slaughterhouse, Iran. Under sterile settings, the protoscoleces were aspirated and transferred into the glass cylinders, which were left for 30 min to let the protoscoleces settle to the bottom of the cylinder. Then the protoscoleces were washed with phosphate-buffered saline (PBS, pH ~ 7.4) three times (three mins for each), and they were set to 1 × 10^3^ protoscoleces per mL [20].

### 2.4. Viability Test

The rate of viability of the obtained protoscoleces was calculated by flame cell mobility as well as vital eosin staining (Sigma-Aldrich, Burlington, MA, USA) solution 0.1% (1 g of eosin powder in 1000 mL distilled water). Five minutes after exposure to the eosin stain, viable and dead parasites appeared with no color and pink, respectively [21].

### 2.5. In Vitro Protoscolicidal Effects

For the in vitro protoscolicidal study, protoscoleces (1 mL) were separately exposed to 1 mL Au-NCs at 100–400 μg/mL in test tubes. The tube contents were gently mixed and then incubated at 37 °C for 10, 20, 30, and 60 min. At the end of each incubation time, the supernatant was discarded and 0.05 mL of eosin stain (0.1%) was added to the mixture. The smears were provided, and the mortality rate of the treated protoscoleces was observed under a light microscope, whereas the viable and dead parasites appeared without color and pink, respectively. Silver nitrate and normal saline as the solvent were applied as the positive and negative drugs, respectively [22].

### 2.6. Ex Vivo Protoscolicidal Effects of Au-NCs

To study the ex vivo properties of Au-NPs on protoscoleces, almost half of the contents of the cyst were discarded, and then Au-NCs at 100–400 μg/mL were inoculated among the cyst to seal the entire cyst. Next, the parasites were harvested in 5–60 min, and the rate of viability was reported based on the method described above [23].

### 2.7. Evaluation of Caspase-3 Activity of Au-NCs on Protoscoleces

Following the treatment of protoscoleces with Au-NCs, the RNA was extracted using the Plus Kit of CinnaGen Co., Tehran, Iran. Then, complementary DNA (cDNA) was produced based on the kit of CinnaGen Co., Iran, using 1 μg of total RNA via hexamer primer (Table 1). Real-time PCR was carried out with a primary denaturation phase at 95 °C for 5 min and then 36 cycles at 94 °C, 57 °C for 40 s, and 74 °C for 40 s. The gene expression level was studied by measuring the ∆ΔCt-2 through CA, Hercules, and Rad-Bio optical system software (iQTM5). To increase the validity of the test, the process was repeated three times, whereas β-actin as a housekeeping gene was utilized to standardize the expression levels of specimens [24].

### 2.8. Effect of Au-NCs on Ultra-Structural Changes in Protoscoleces

To study the effect of Au-NCs on ultra-structural changes in protoscoleces, after fixing the treated parasites in 2.5% glutaraldehyde at room temperature for 4 h, the protoscoleces were washed using PBS. After washing the fixed protoscoleces, the process of dewatering began in rising grades of alcohol from 50 to 100% *v*/*v* for 15 min. Then, the protoscoleces stretched the final level of drying through a dryer device (S4160, Hitachi, Tokyo, Japan) for 30 min. The exposed protoscoleces were then fixed and coated in gold using an SEM coating device (E5100 Polaron, Watford, UK). To finish, the samples were studied under scanning electron microscopy (SEM, JOEL 64000, Tokyo, Japan) at a 15–25 KV voltage, magnification of 10×, and resolution of 1 nm [25].

### 2.9. Cytotoxicity Effects of Au-NCs

#### 2.9.1. Cell Culture

To test the cytotoxicity of Au-NCs, hepatocellular carcinoma (HepG2) and normal embryonic kidney (HEK293) cell lines (Pasteur Institute of Iran) were cultivated in Dulbecco’s Modified Eagle Medium (DMEM) upgraded with pen-strep (100 μg/mL) and fetal bovine serum (FBS) at 37 °C in 5% CO_2_. 

#### 2.9.2. Cell Viability Assay

The cytotoxicity procedure was carried out based on previous studies [26]. In the first step, normal and cancer cells (1 × 10^5^/mL) were incubated with Au-NCs (10–400 μg/mL) in culture 96-well plates at 24 °C for 48 h. Following the pouring of the MTT (3-[4,5-dimethylthiazol-2-yl]-2,5-diphenyl tetrazolium bromide) suspension (5 mg/mL, Sigma-Aldrich, Germany), the plate was again kept warm for 4 h. In the last step, DMSO as the stopping agent was added, the mixture was left for 30 min, and the optical density of the plates was recorded by the ELISA reader at 570 nm. The cytotoxicity was measured by reporting the cytotoxic concentration of 50% (CC_50_) using Probit examination in SPSS software ver. 26.0 [26]. 

#### 2.9.3. Selectivity Index (SI)

To exhibit the selective cytotoxicity of the green synthesized Au-NCs on normal and cancer cells, the SI value was obtained through the equation of CC_50_ of normal cells on CC_50_ of cancer cells. 

### 2.10. Data Analysis 

As mentioned above, the tests were performed in triplicate. One-way analysis of variance and post hoc tests in SPSS software ver. 26.0 were applied to the data analysis to compare the differences between groups. The significant level was also shown as *p* < 0.05.

## 3. Results

### 3.1. Biosynthesis and Compositional Characterization of the Au-NCs

**Figure 1:**
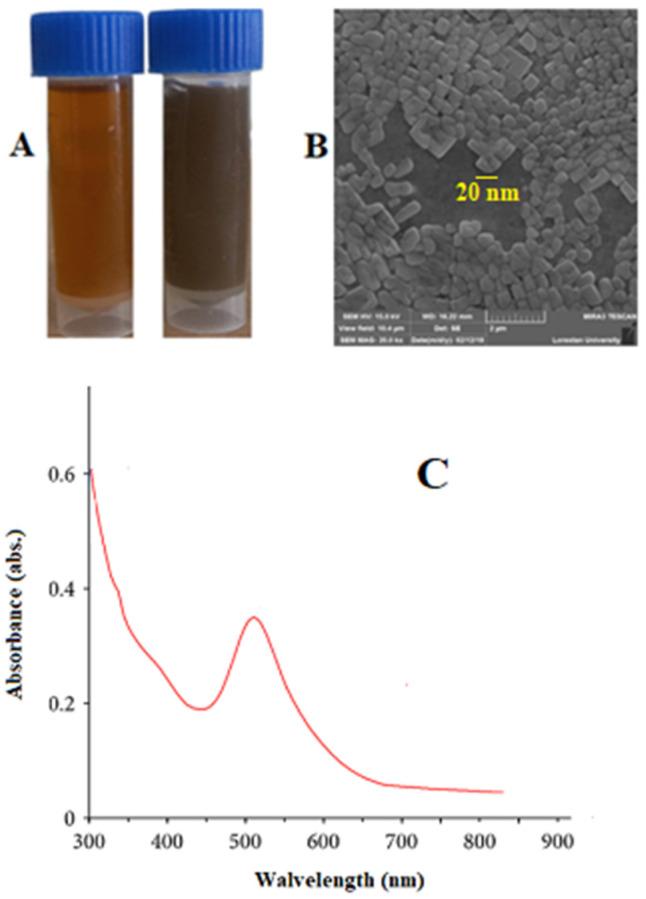
(**A**) Synthesized gold nanoparticles (Au-NCs), (**B**) scanning electron microscope image, and (**C**) UV-vis spectroscopic analysis of synthesized Au-NCs.

Figure 1A exhibits the equality, proper distribution, and homogeneity of Au-NCs. The quick decrease of the Au ions via *S. khuzestanica* extract caused the homogeneous nucleation of Au that resulted in the production of Au-NCs. The Au-NCs are restrained inside a surrounding substance of extract, which probably contains biomolecules that are stabilizers or covering causes in synthesis. The synthesized Au-NCs are cubes and have an average size of 20–30 nm (Figure 1B). The absorption peak of nanoparticles obtained from the Vis-UV was observed at 511 nm, indicating the presence of silver nanoparticles.

**Figure 2:**
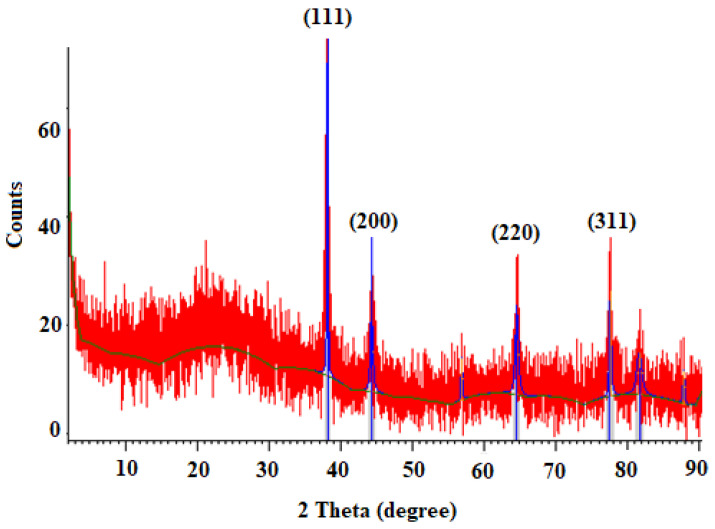
X-ray diffraction (XRD) pattern of synthesized gold nanoparticles (Au-NCs).

Figure 2 exhibits the XRD profile of green synthesized Au NPs. The diffraction peaks at 2*θ* 38.1564.61°, 44.33°, and 77.62° are related to the (111), (200), (220), and (311) Bragg planes of face-centered cubic (FCC) and gold lattice, respectively, that are in line with the typical diffraction of gold (JCPDS80-3697).

**Figure 3:**
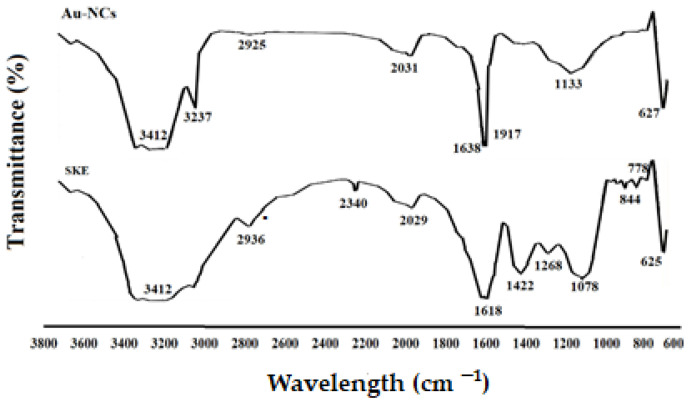
Fourier transforms infrared spectroscopy spectra for *Saturja khuzistanica* (SKE) and gold nanoparticles (Au-NCs).

The attendance of secondary metabolites, which are accountable for the covering and lessening of the HAuCl4 to Au-NCs, was examined by FTIR examination. As shown in Figure 3, the same FTIR spectrums of the extract and Au-NCs, confirmed the green synthesis of Au-NCs. The attendance of several IR bands is associated to the presence of various functional groups in the extract. The peaks at 1078, 1268, 3412, and 2936 cm^−1^ are linked to C–O, C–O–C (oligosaccharides or in triacylglycerols), O–H, and C–H (carbohydrates) stretching, respectively. While the peaks in the range 1618–2029 cm^−1^ were probably linked to the saturated hydrocarbons (Csp3–H), C=C, and C=O stretching.

### 3.2. In Vitro Protoscolicidal Effects of Au-NCs

**Figure 4:**
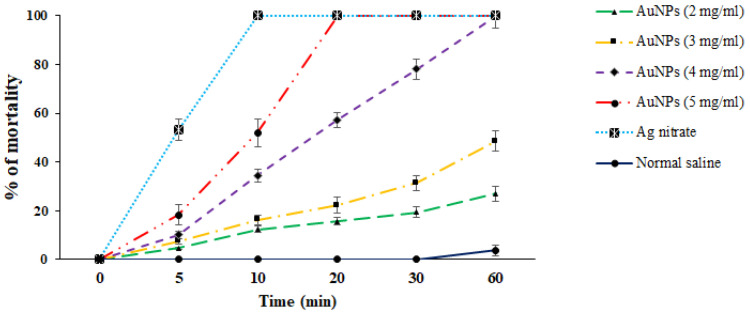
In vitro effects of green synthesized gold nanoparticles (Au-NCs) and silver nitrate (Ag-nitrate) against hydatid cyst protoscoleces (*n* = 3).

In vitro protoscolicidal effects of green synthesized Au-NCs were illustrated in Figure 4. The findings discovered that the scolicidal efficacy of Au-NCs was dose-time-dependently increased (*p* < 0.001). Between the concentrations of Au-NCs, the main scolicidal efficacy was observed at 5 mg/mL with 100% mortality after 20 min of treatment to protoscoleces. Concerning other concentrations, the rate of mortality for 2, 3, and 4 mg/mL were reported as 27.1%, 48.6%, and 100% after 60 min, respectively. The viability rate of protoscoleces in the normal saline and Ag-nitrate was 96.3 and 0% after 60 and 10 min, respectively (Figure 4). 

### 3.3. Ex Vivo Protoscolicidal Effects of Au-NCs

**Figure 5:**
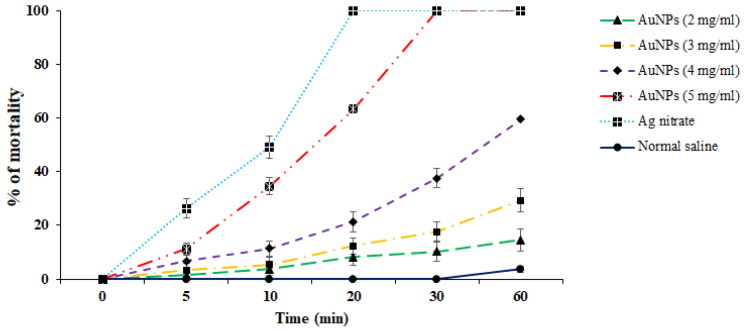
Ex vitro effects of the green synthesized gold nanoparticles (Au-NCs) and silver nitrate (Ag-nitrate) against hydatid cyst protoscoleces (*n* = 3).

The ex vivo results indicated that, in parallel to the in vitro assay, Au-NCs displayed promising scolicidal effects against hydatid cyst protoscoleces. However, Au-NCs needed further exposure time to indicate more protoscolicidal effects (Figure 5). The assay showed that Au-NCs at concentrations of 4 and 5 µg/mL revealed their protoscolicidal effects with 59 and 100% mortality after 60 and 30 min of exposure, respectively.

### 3.4. Evaluation of Caspase-3 Activity of Au-NCs on Protoscoleces

**Figure 6:**
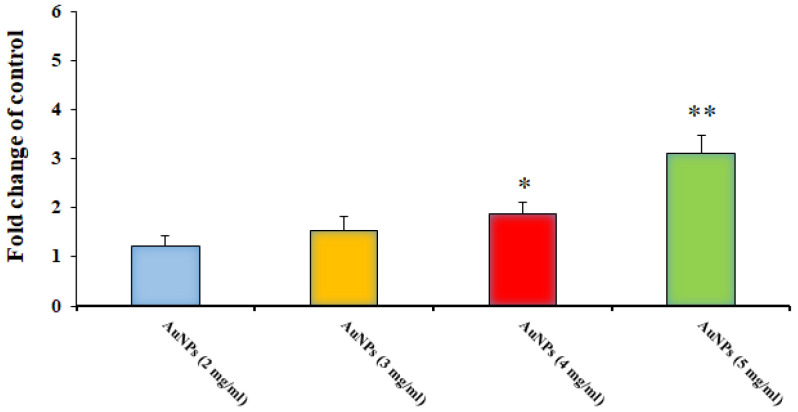
Effect of the green synthesized gold nanoparticles on the expression level of the Caspase-3 gene in hydatid cyst protoscoleces (*n* = 3). * *p* < 0.001 and ** *p* < 0.01.

As shown in Figure 6, by real-time PCR, we found that after treatment of protoscoleces with Au-NCs, the gene of caspase-3 was dose-dependently upregulated compared with the control group (*p* < 0.05), so that Au-NCs at 4 and 5 mg/mL significantly upregulated the expression level of the caspase-3 gene.

### 3.5. Effect of Au-NCs on Ultra-Structural Changes in Protoscoleces

**Figure 7:**
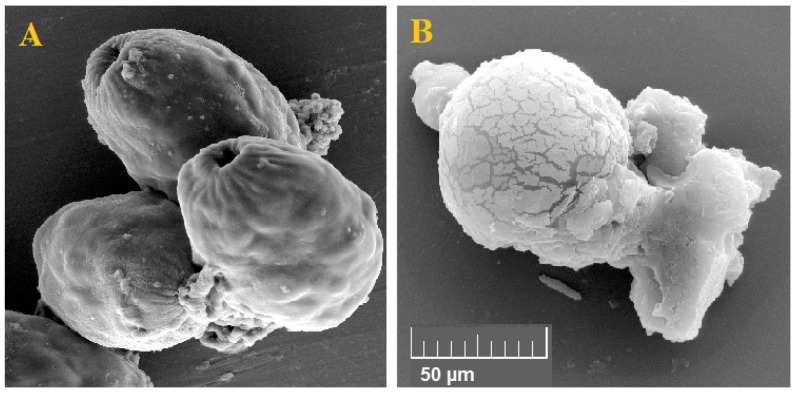
Effect of the green synthesized gold nanoparticles on the exterior ultrastructural effects of hydatid cyst protoscoleces through scanning electron microscopy. Non-treated (**A**) and treated (**B**) protoscoleces.

The findings of SEM showed that in non-treated protoscoleces, smoothness and integrity, while in protoscoleces treated with Au-NCs at 5 mg/mL) weakening and disintegration of the cell wall, wrinkles, and protrusions due to the formation of blebs were observed (Figure 7).

### 3.6. Cytotoxicity Effects of Au-NCs

The outcomes of the MTT analysis displayed that Au-NCs dose-dependently declined the cell viability of HepG_2_ and HEK293 cells with CC50 values of 97.4 and 200.2 μg/mL, respectively, whereas the obtained SI was 2.05.

## 4. Discussion

Hydatid cyst is a parasitic and common disease among humans and animals initiated by the larvae of a canine intestinal parasite in humans and animals called *E. granulosus* [8]. In humans and other intermediate hosts, after swallowing the egg, any organ of the body can be the site of cyst formation, and depending on which organ the cyst is in, we have organ dysfunction. Lung and liver cysts are usually more common, whereas the signs of the illness also depend on the involved organ, the cyst size, the rupture of the cyst, and subsequent immunological reactions such as anaphylactic shock [9]. The incubation period may last between 5 and 20 years, and in most cases, it takes years for the disease to become symptomatic, and sometimes the cyst heals on its own. After years, due to its enlargement and pressure effect, the patient feels abdominal pain, loss of appetite, and a feeling of an abdominal mass [10]. Surgery is now the ideal technique and the first option of treatment for symptomatic patients [13]. One of the measures to make cyst surgery safe is the use of suitable scolicidal agents such as the injection of a hypertonic nitrate salt, formalin, hypertonic saline, silver nitrate, and cetrimide into the cyst [15]. Unfortunately, most of these scolicidal substances that are injected inside cysts have side effects, including leaking out of the cyst and adverse effects on the living tissue of the host, such as necrosis of liver cells, which limits their use [16,17]. Hence, we studied the lethal effect of gold nanoparticles green synthesized with an aqueous extract of *S. khuzestanica*, a medicinal herb with various biological properties, so that if favorable results are observed, it can be recommended for use before cyst surgery. Here, we found that the synthesized Au-NCs are cubes and have an average size of 20–30 nm. In infrequent cases, some synthesized Au-NCs displayed larger sizes; however, the most frequent size for green synthesized Au-NCs was reported to range from 15 to 50 nm. By XRD pattern, we observed that the diffraction peaks at 1078, 1268, 3412, and 2936 cm^−1^ are related to the (111), (200), (220), and (311) Bragg planes of face-centered cubic (FCC) and gold lattice, respectively, that are in line with the typical diffraction of gold (JCPDS80-3697). In FTIR analysis, the presence of several IR bands is associated with the presence of various functional groups in the extract. The peaks at 1078, 1268, 3412, and 2936 cm^−1^ are linked to C–O, C–O–C (oligosaccharides or in triacylglycerols), O–H, and C–H (carbohydrates) stretching, respectively. While the peaks in the range 1618–2029 cm^−1^ were probably linked to the saturated hydrocarbons (Csp3–H), C=C, and C=O stretching. These reported peaks might be measured to indicate the presence of some compounds, e.g., phenolic, flavonoid, and carboxylic [27]. In vitro protoscolicidal effects revealed that the scolicidal efficacy of Au-NCs was dose-time-dependently increased (*p* < 0.001), whereas the main scolicidal efficacy was observed at 5 mg/mL with 100% mortality after 20 min of treatment with protoscoleces. The ex vivo results indicated that, in parallel to the in vitro assay, Au-NCs displayed promising scolicidal effects against hydatid cyst protoscoleces, but Au-NCs needed further exposure time to indicate more protoscolicidal effects. The assay showed that Au-NCs at concentrations of 4 and 5 µg/mL revealed their protoscolicidal effects with 59 and 100% mortality after 60 and 30 min of exposure, respectively. 

At present, the synthesis of Au-NCs is of high interest for investigation in detection methods and drug development of several pathogenic parasite strains, e.g., *Toxoplasma gondii*, *Plasmodium* spp., *Trypanosoma* spp., *Leishmania* spp., *Schistosoma* spp., *Enterocytozoon hepatopenaei*, and *Cryptosporidium parvum*, letting scientists consider Au-NCs as a new agent for drug development [28]. Considering the protoscolicidal effects of green synthesized Au-NCs, Çolak et al. (2019) showed that synthesized photothermal Au-NCs, mainly at a dose of 0.8 mL after 120 min of incubation, eliminated 89.3% of hydatid cyst protoscoleces in vitro [29]. Barabadi et al. (2017) reported that Au-NCs, a green chemical synthesized by *Penicillium aculeatum,* had considerable in vitro scolicidal effects, especially at 0.3 mg/mL, so that it eliminated more than 90% of protoscoleces after 120 min of exposure [30]. These differences were probably due to some factors, such as the method of Au-NCs synthesis and the time of exposure. As the antimicrobial mechanisms of action of Au-NCs, previous studies reported that these NPs displayed antiparasitic effects by stopping the trypanothione reductase in *Leishmania* parasites, inhibiting the cysteine protease falcipain-2 enzyme in *Plasmodium* parasites, triggering the reactive oxygen species (ROS) as well as the interaction and entry into microbes, and disturbing the protein synthesis and replication of DNA [31,32,33].

By real-time PCR, we found that after treatment of protoscoleces with Au-NCs, the gene for caspase-3 was dose-dependently upregulated compared with the control group (*p* < 0.05). In agreement with our results, Baharara et al. (2016) reported that Au-NCs green synthesized by *Zataria multiflora* extract showed potent cytotoxic effects against human cervical cancer cells through apoptosis induction via triggering of Caspase-3 and 9 [34]. Liu et al. (2019) revealed that Au-NCs green synthesized by *Curcuma wenyujin* extract had considerable cytotoxic effects against human renal cancer cells by elevating the level of Caspase 3, 9, Bid, and Bad and declining the level of Bcl-2 and Bcl-xl genes [35]. Therefore, these findings showed that apoptosis induction can be considered one of the protoscolicidal mechanisms of green-synthesized Au-NCs. 

By SEM, we reported that in non-treated protoscoleces, smoothness and integrity were observed while in protoscoleces treated with Au-NCs at 5 mg/mL, weakening and disintegration of the cell wall, wrinkles, and protrusions due to the formation of blebs were observed. Previous studies exhibited that in Au-NCs, Au+ ions through interaction with SH groups of membrane and organelles of cells result in coagulation of proteins, disturbing the production of the cell wall, ergosterol pathway inhibition in the cell wall, interference of microbial breathing, and hindering of main transport chains [36,37,38,39,40]. Hence, it was suggested that direct effects on the cell wall and critical organelles may be one of the main protoscolicidal mechanisms of Au-NCs. The outcomes of the MTT analysis displayed that Au-NCs dose-dependently declined the cell viability of HepG_2_ and HEK293 cells with CC_50_ values of 97.4 and 200.2 μg/mL, respectively. The SI was calculated at 2.05 (>2), indicating that Au-NCs are safe for normal cells rather than cancer cells. The main limitations of the present study are the lack of toxicity and the lack of some mechanisms of action of AuNPs, such as their potential impact on the immune system.

## 5. Conclusions

The findings of the current experimental investigation reported the effective in vitro and ex vivo scolicidal effects of green synthesized AuNPs against hydatid cyst protoscoleces. We also evaluated the possible mechanisms of these nanoparticles by assessing their effects on the cell wall of the protoscoleces as well as their effects on provoking the apoptosis process of caspase-3 activation. As a positive point in this study, we found that green synthesized gold nanoparticles had no significant cytotoxicity against human normal cells. With all these descriptions, it is suggested that additional studies be conducted in vivo and experimentally on animals to determine the exact effective concentration and its possible harmful side effects on the internal organs of the body should also be investigated so that the obtained results can be applied. 

## Figures and Tables

**Table 1:** The primer sequence for used genes in real-time PCR.

Primer	Sequence
Caspase-3	F: 5′ TTCATTATTCAGGCCTGCCGAGG-3′ R: 5′-TTCTGACAGGCCATGTCATCCTCA-3
β-actin	F: GTGACGTTGACATCCGTAAAGA R: GCCGGACTCATCGTACTCC

## Data Availability

All data generated or analyzed during this study are included in this published article.

## References

[1] Emerich D.F., Thanos C.G. (2003). Nanotechnology and medicine. Expert Opin. Biol. Ther..

[2] Nejati K., Dadashpour M., Gharibi T., Mellatyar H., Akbarzadeh A. (2021). Biomedical Applications of Functionalized Gold Nanoparticles: A Review. J. Clust. Sci..

[3] Hasan S. (2015). A review on nanoparticles: Their synthesis and types. Res. J. Recent Sci..

[4] Roy A., Kunwar S., Bhusal U., Alghamdi S., Almehmadi M., Alhuthali H.M., Allahyani M., Hossain J., Hasan A., Sarker M.R. (2023). Bio-Fabrication of Trimetallic Nanoparticles and Their Applications. Catalysts.

[5] Rotti R.B., Sunitha D.V., Manjunath R., Roy A., Mayegowda S.B., Gnanaprakash A.P., Alghamdi S., Almehmadi M., Abdulaziz O., Allahyani M. (2023). Green synthesis of MgO nanoparticles and its antibacterial properties. Front. Chem..

[6] Gour A., Jain N.K. (2019). Advances in green synthesis of nanoparticles. Artif. Cells Nanomed. Biotechnol..

[7] Teimuri-Mofrad R., Hadi R., Tahmasebi B., Farhoudian S., Mehravar M., Nasiri R. (2017). Green synthesis of gold nanoparticles using plant extract: Mini-review. Nanochem. Res..

[8] Moro P., Schantz P.M. (2009). Echinococcosis: A review. Int. J. Infect. Dis..

[9] Eckert J., Thompson R. (2017). Historical Aspects of Echinococcosis. Adv. Parasitol..

[10] Hemphill A., Rufener R., Ritler D., Dick L., Lundström-Stadelmann B. (2019). Drug Discovery and Development for the Treatment of Echinococcosis, Caused by the Tapeworms Echinococcus granulosus and Echinococcus multilocularis. Negl. Trop. Dis. Drug Discov. Dev..

[11] Mahmoudvand H., Mahmoudvand H., Oliaee R.T., Kareshk A.T., Mirbadie S.R., Aflatoonian M.R. (2017). In vitro protoscolicidal effects of Cinnamomum zeylanicum essential oil and its toxicity in mice. Pharmacog. Mag..

[12] Stojkovic M., Zwahlen M., Teggi A., Vutova K., Cretu C.M., Virdone R., Nicolaidou P., Cobanoglu N., Junghanss T. (2009). Treatment response of cystic echinococcosis to benzimidazoles: A systematic review. PLoS Negl. Trop Dis..

[13] Velasco-Tirado V., Alonso-Sardón M., Lopez-Bernus A., Romero-Alegría Á., Burguillo F.J., Muro A., Carpio-Pérez A., Bellido J.L., Pardo-Lledias J., Cordero M. (2018). Medical treatment of cystic echinococcosis: Systematic review and meta-analysis. BMC Infect. Dis..

[14] Junghanss T., Brunetti E., Chiodini P.L., Horton J., da Silva A.M. (2008). Clinical Management of Cystic Echinococcosis: State of the Art, Problems, and Perspectives. Am. J. Trop. Med. Hyg..

[15] Rajabi M.A. (2009). Fatal reactions and methaemoglobinaemia after silver nitrate irrigation of hydatid cyst. Surg. Pr..

[16] Albalawi A.E., Alanazi A.D., Baharvand P., Sepahvand M., Mahmoudvand H. (2020). High Potency of Organic and Inorganic Nanoparticles to Treat Cystic Echinococcosis: An Evidence-Based Review. Nanomaterials.

[17] Jafari F., Ghavidel F., Zarshenas M.M. (2016). A Critical Overview on the Pharmacological and Clinical Aspects of Popular Satureja Species. J. Acupunct. Meridian Stud..

[18] Sidorowicz A., Margarita V., Fais G., Pantaleo A., Manca A., Concas A., Rappelli P., Fiori P.L., Cao G. (2022). Characterization of nanomaterials synthesized from Spirulina platensis extract and their potential antifungal activity. PLoS ONE.

[19] Maqbool Q., Yigit N., Stöger-Pollach M., Ruello M.L., Tittarelli F., Rupprechter G. (2023). *Operando* monitoring of a room temperature nanocomposite methanol sensor. Catal. Sci. Technol..

[20] Niazi M., Saki M., Sepahvand M., Jahanbakhsh S., Khatami M., Beyranvand M. (2019). In vitro and ex vivo scolicidal effects of Olea europaea L. to inactivate the protoscolecs during hydatid cyst surgery. Ann. Med. Surg..

[21] Mahmoudvand H., Pakravanan M., Aflatoonian M.R., Khalaf A.K., Niazi M., Mirbadie S.R., Kareshk A.T., Khatami M. (2019). Efficacy and safety of Curcuma longa essential oil to inactivate hydatid cyst protoscoleces. BMC Complement. Altern. Med..

[22] Mahmoudvand H., Pakravanan M., Kheirandish F., Jahanbakhsh S., Sepahvand M., Niazi M., Rouientan A., Aflatoonian M.R. (2020). Efficacy and Safety *Curcuma zadoaria* L. to Inactivate the Hydatid Cyst Protoscoleces. Curr. Clin. Pharmacol..

[23] Shakibaie M., Khalaf A.K., Rashidipour M., Mahmoudvand H. (2022). Effects of green synthesized zinc nanoparticles alone and along with albendazole against hydatid cyst protoscoleces. Ann. Med. Surg..

[24] Ezzatkhah F., Khalaf A.K., Mahmoudvand H. (2021). Copper nanoparticles: Biosynthesis, characterization, and protoscolicidal effects alone and combined with albendazole against hydatid cyst protoscoleces. Biomed. Pharmacother..

[25] Cheraghipour K., Azarhazine M., Zivdari M., Beiranvand M., Shakib P., Rashidipour M., Mardanshah O., Mohaghegh M.A., Marzban A. (2023). Evaluation of scolicidal potential of salicylate coated zinc nanoparticles against Echinococcus granulosus protoscoleces. Exp. Parasitol..

[26] Albalawi A.E., Khalaf A.K., Alyousif M.S., Alanazi A.D., Baharvand P., Shakibaie M., Mahmoudvand H. (2021). Fe3O4@piroctone olamine magnetic nanoparticles: Synthesize and therapeutic potential in cutaneous leishmaniasis. Biomed. Pharmacother..

[27] Maqbool Q., Czerwinska N., Giosue C., Sabbatini S., Ruello M.L., Tittarelli F. (2022). New waste-derived TiO_2_ nanoparticles as a potential photocatalytic additive for lime based indoor finishings. J. Clean. Prod..

[28] Benelli G. (2018). Gold nanoparticles–against parasites and insect vectors. Acta Trop..

[29] Çolak B., Aksoy F., Yavuz S., Demircili M.E. (2019). Investigating the effect of gold nanoparticles on hydatid cyst protoscolices under low-power green laser irradiation. Turk. J. Surg..

[30] Barabadi H., Honary S., Mohammadi M.A., Ahmadpour E., Rahimi M.T., Alizadeh A., Naghibi F., Saravanan M. (2017). Green chemical synthesis of gold nanoparticles by using Penicillium aculeatum and their scolicidal activity against hydatid cyst protoscolices of Echinococcus granulosus. Environ. Sci. Pollut. Res..

[31] Colotti G., Ilari A., Fiorillo A., Baiocco P., Cinellu M.A., Maiore L., Scaletti F., Gabbiani C., Messori L. (2013). Metal-based compounds as prospective antileishmanial agents: Inhibition of trypanothione reductase by selected gold complexes. Chem. Med. Chem..

[32] Micale N., Cinellu M.A., Maiore L., Sannella A.R., Severini C., Schirmeister T., Gabbiani C., Messori L. (2011). Selected gold compounds cause pronounced inhibition of Falcipain 2 and effectively block P. falciparum growth in-vitro. J. Inorg. Biochem..

[33] Heinlaan M., Ivask A., Blinova I., Dubourguier H.C., Kahru A. (2008). Toxicity of nanosized and bulk ZnO, CuO and TiO2 to bacteria Vibrio fisceri and crustaceans Daphnia magna and Thamnocephalus platyurus. Chemosphere.

[34] Baharara J., Ramezani T., Divsalar A., Mousavi M., Seyedarabi A. (2016). Induction of Apoptosis by Green Synthesized Gold Nanoparticles Through Activation of Caspase-3 and 9 in Human Cervical Cancer Cells. Avicenna J. Med. Biotechnol..

[35] Liu R., Pei Q., Shou T., Zhang W., Hu J., Li W. (2019). Apoptotic effect of green synthesized gold nanoparticles from Curcuma wenyujin extract against hsuman renal cell carcinoma A498 cells. Int. J. Nanomed..

[36] Slavin Y.N., Asnis J., Hńfeli U.O., Bach H. (2017). Metal nanoparticles: Understanding the mechanisms behind antibacterial activity. J. Nanobiotechnol..

[37] Wang L., Hu C., Shao L. (2017). The antimicrobial activity of nanoparticles: Present situation and prospects for the future. Int. J. Nanomed..

[38] Li Y., Zhang W., Niu J., Chen Y. (2012). Mechanism of photogenerated reactive oxygen species and correlation with the antibacterial properties of engineered metal-oxide nanoparticles. ACS Nano.

[39] Mahmoudvand H., Kheirandish F., Ghasemi Kia M., Tavakoli Kareshk A., Yarahmadi M. (2016). Chemical composition, protoscolicidal effects and acute toxicity of Pistacia atlantica Desf. fruit extract. Nat. Prod. Res..

[40] Mohamed A.E., Elgammal W.E., Eid A.M., Dawaba A.M., Ibrahim A.G., Fouda A., Hassan S.M. (2022). Synthesis and characterization of new functionalized chitosan and its antimicrobial and in-vitro release behavior from topical gel. Int. J. Biol. Macromol..

